# A coding-complete genome sequence of bovine-like coronavirus identified in white-tailed deer (*Odocoileus virginianus*) in the United States

**DOI:** 10.1128/mra.00500-26

**Published:** 2026-07-10

**Authors:** Hannah Cluett, Jeffrey C. Chandler, Bledar Bisha

**Affiliations:** 1 Department of Animal Science, University of Wyoming 4416, Laramie, Wyoming, USA; 2 Wildlife Disease Diagnostic Laboratory, Wildlife Services, U.S. Animal and Plant Health Inspection Services, U.S. Department of Agriculture, Fort Collins, Colorado, USA; DOE Joint Genome Institute, Berkeley, California, USA

**Keywords:** coronavirus, metagenomics, deer

## Abstract

Bovine coronavirus within the *Embecovirus* subgenus causes respiratory and enteric diseases in domestic cattle. We report a coding-complete genome of bovine-like coronavirus from a white-tailed deer (*Odocoileus virginianus*) in New Jersey, USA. This genome is 30,988 bp with a guanine-cytosine content of 37%.

## ANNOUNCEMENT

Bovine coronavirus (BCoV) within subgenus *Embecovirus* (genus *Betacoronavirus*, family Coronaviridae) is a pneumoenteric, single-stranded RNA virus associated with disease in cattle (1). The bovine-like coronaviruses reported in wild and captive ruminants share 98–99% nucleic acid identity with BCoVs (2, 3). Despite ongoing evidence of bovine-like coronavirus exposure in cervids, genome sequences from white-tailed deer (*Odocoileus virginianus*) remain limited (4). The most recent publicly available BCoV genome from a white-tailed deer was collected in 1994 (5). Here, we describe a coding-complete bovine-like coronavirus genome from a free-ranging white-tailed deer nasal swab collected in Cumberland County, New Jersey.

The nasal swab was collected from a hunter-harvested white-tailed deer in 2022 by United States Department of Agriculture Wildlife Services and stored in PrimeStore molecular transport media (Longhorn Diagnostics, USA). Total nucleic acid was extracted using the QIAamp MinElute Virus Spin Kit (Qiagen, Germany) and eluted in 75 μL following the manufacturer’s guidelines. Libraries were prepared from 50 ng of nucleic acid with Illumina’s RNA with Enrichment Prep (Illumina, USA). Probe-based hybridization capture was performed using 200 ng of library input with Illumina Respiratory Virus Enrichment Panel’s v2 oligos. The library was sequenced on an Illumina NextSeq2000 using a 100 bp paired-end read format.

For bioinformatic analysis, default parameters were used unless otherwise specified. The quality of the 1.5 M paired raw reads was assessed by FastQC v0.12.0 (6). Host reads were removed using Hostile v1.1.0 (7) against reference genome GCA_023699985.4, resulting in 930,000 paired reads. Adapter and low-quality bases were removed using Trimmomatic v0.39 (8). *De novo* assembly and scaffolding were performed by coronaSPAdes v4.0.0 (9), generating 438 contigs (36–29,361 bp). A total of 199 contigs (mean 588 bp) remained after filtering out contigs <200 bp. The two coronavirus-associated contigs (29,631 bp and 1,672 bp) resulted in a 30,998 bp genome post-scaffolding. No additional genome polishing was performed.

The genome was taxonomically screened by Kraken2 v2.1.3 (10) and annotated by geNomad v1.11.0 (11). Annotations were verified using BLASTn and BLASTp against the National Center for Biotechnology Information (NCBI) core nucleotide and non-redundant protein databases (NCBI Online BLAST Dec. 2025) (12). The coding-complete genome had an average depth of 6,300× and a guanine-cytosine content of 37%. Genome termini were not experimentally validated; however, all canonical coding sequences were recovered. Comparative analysis using BLASTn demonstrated >98% nucleotide identity to BCoV. Protein annotations identified 10 coding regions with high amino acid similarity to BCoV (Table 1). The ongoing identification of bovine-like coronaviruses in white-tailed deer underscores the need for continued surveillance to better understand its distribution in North American cervids.

**TABLE 1:** Protein similarity of the recovered bovine-like coronavirus (PZ105977) to BCoV (OR502443.1) and a bovine-like coronavirus isolated from WTD in 1994 (GCA_031121515.1)

Annotation	Total size (AA)	% Identity to BCoV	% Identity to WTD BCoV ‘94
Orf1ab polyprotein	7,082	99.53	99.23
32kDa protein	278	99.64	98.20
Hemagglutinin-esterase	424	99.06	99.29
Spike	1,363	99.41	98.68
4.9 kDa nsp	29	100.0	N/A^*a*^
4.8 kDa nsp	41	100.0	92.31
12.7 kDa nsp	109	100.0	99.08
Envelope	169	100.0	100.00
Membrane	230	100.0	98.70
Nucleocapsid	448	99.78	99.55

^*a*^ N/A, not applicable.

## Data Availability

The coding-complete sequence of the detected bovine-like coronavirus is deposited in GenBank under accession no. PZ105977. Raw reads have been deposited in NCBI’s SRA under accession SRX32467364 within BioProject PRJNA1426439.
